# Rapid Screening of Proanthocyanidins from the Roots of *Ephedra sinica* Stapf and its Preventative Effects on Dextran-Sulfate-Sodium-Induced Ulcerative Colitis

**DOI:** 10.3390/metabo12100957

**Published:** 2022-10-10

**Authors:** Mengying Lv, Yang Wang, Xiayun Wan, Bo Han, Wei Yu, Qiaoling Liang, Jie Xiang, Zheng Wang, Yanqing Liu, Yayun Qian, Fengguo Xu

**Affiliations:** 1 Department of Pharmacy, Institute of Translational Medicine, Medical College, Yangzhou University, Yangzhou 225001, China; 2 The Key Laboratory of Syndrome Differentiation and Treatment of Gastric Cancer of the State Administration of Traditional Chinese Medicine, Yangzhou 225001, China; 3 Key Laboratory of Xinjiang Phytomedicine Resource and Utilization, Department of Pharmacy, Ministry of Education, Shihezi University, Shihezi 832002, China; 4 Department of Pathology, Affiliated Hospital of Yangzhou University, Yangzhou 225001, China; 5 Key Laboratory of Drug Quality Control and Pharmacovigilance, Ministry of Education, China Pharmaceutical University, Nanjing 210009, China

**Keywords:** proanthocyanidins, root of *Ephedra sinica*, the ethyl acetate extract, dextran-sulfate-sodium-induced ulcerative colitis, UPLC-LTQ-orbitrap mass spectrometry

## Abstract

Proanthocyanidins (PACs) have been proven to exert antioxidant and anti-inflammatory effects. In this study, ultra-performance liquid chromatography (UPLC) coupled with linear ion trap-Orbitrap (LTQ-Orbitrap) high-resolution mass spectrometry was first employed to systematically screen PACs from the roots of *Ephedra sinica* Stapf, and its ethyl acetate extract (ERE) was found to contain PAC monomers and A-type dimeric proanthocyanidins, which were tentatively identified through characteristic fragmentation patterns. In vitro, the antioxidant activity of ERE was tested through 2,2-diphenyl-1-picrylhydrazyl (DPPH) and 2, 2-azino-bis-3-ethylbenzothiazoline-6-sulfonic acid (ABTS) assays. In addition, ERE could inhibit the production of nitric oxide (NO) in lipopolysaccharide (LPS)-induced RAW 264.7 cells. In vivo, the preventative effects on dextran-sulfate-sodium-induced ulcerative colitis in mice was investigated. Mice were administered with ERE for 21 days, and during the last 7 days of the treatment period dextran sulfate sodium (DSS) was used to induce experimental colitis. The results showed that ERE treatment alleviated DSS-induced colitis, which was characterized by decreases in disease activity index (DAI) scores, spleen index and colon levels of TNF-α and IL-6, mitigation in pathological damage and oxidative stress and increases in colon length and IL-10 levels. In conclusion, supplementation of PACs derived from ERE may offer a new strategy for the treatment of ulcerative colitis. Moreover, our research will greatly facilitate better utilization of Ephedra plants.

## 1. Introduction

Ulcerative colitis (UC) is one of the major types of inflammatory bowel disease (IBD) that is characterized by chronic and recurrent inflammation in the intestinal tissue [1]. The major symptoms of UC are abdominal pain, vomiting, diarrhea, rectal bleeding and weight loss, which greatly decreases the quality of life of UC patients [2]. Although there has been a great endeavor to develop anti-UC drugs over the past twenty years, the remission rate is still at 20–30%, which seems to be confronted with the so-called therapeutic ceiling [3]. Common therapeutic options for UC include 5-aminosalicylates (5-ASA), corticosteroids and biologics targeting tumor necrosis factor (TNF), some of which have side effects such as liver toxicity, allergic reactions and drug resistance [4]. In view of this, increasing attention has been paid to alternative and complementary therapies that focus on the prevention and control of the disease without compromising an individual’s quality of life. Phenolic compounds, or polyphenols, present in various plants have shown promising effects in IBD-related in vitro pre-clinical and clinical research [5].

**Figure 1:**
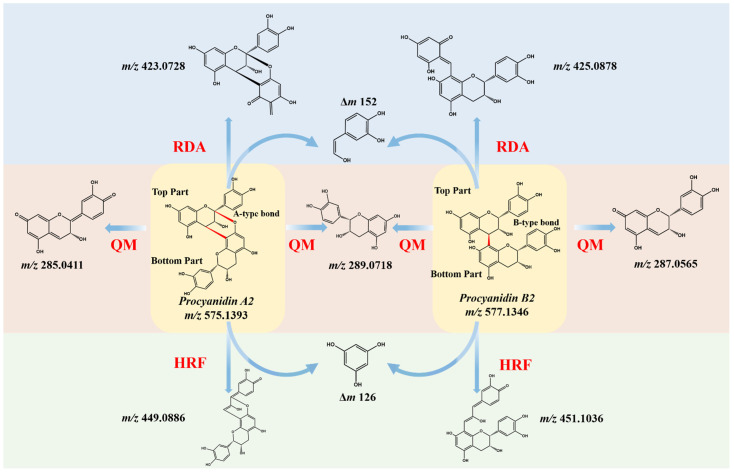
The main fragmentation pathways of Procyanidin dimers with A-type (Procyanidin A2) and B-type (Procyanidin B2) linkages (QM: quinone methide fission, HRF: heterocyclic ring fission; RDA: Retro-Diels-Alder).

The main classes of polyphenols in fruits and plants are flavonoids and non-flavonoid phenolics. Polyphenols or flavonoids have been found to relieve dysregulated inflammatory responses, oxidative stress, or gut microbiome imbalance in ulcerative colitis [6]. Our previous untargeted comparative analysis of the stems and roots of *Ephedra sinica* Stapf (ES) revealed that the roots of ES (RE) contained higher levels of macrocyclic spermine alkaloids and biflavones (also called dimeric proanthocyanidins) compared with the stems of ES [7]. ES belongs to the genus Ephedra of the Ephedraceae family, and the stems of ES have been used to induce perspiration for the treatment of colds; however, RE has been used as an antiperspirant, which reflects an interesting phenomenon in traditional Chinese medicine—same origin, different efficacy [8]. Dimeric proanthocyanidins (PACs) consist of two flavan-3-ol units including (epi)gallocatechin, (epi)catechin and (epi)afzelechin linked through only one single interflavan carbon bond (C_4_-C_8_ or C_4_-C_6_, B-type, Figure 1) or an additional ether bond (C2-O-C7, A-type, Figure 1). Grape seeds, apples and cocoa are good sources for isolating B-type PACs, and A-type PACs can be found in cranberries, peanut skins and cinnamon [9].

Although the pathophysiology of UC is still elusive, oxidative stress has been an important factor in UC progression, as the overproduction of reactive oxygen species (ROS) may cause oxidative damage to cellular biomolecules [10]. PACs contain many hydroxyl groups, which could undergo redox reactions, thus scavenging free radicals. Notably, the best advantage of PACs’ free radical scavenging activity is its ability to be absorbed onto the surface of cell membranes [11]. It has been widely reported that the grape seed, cranberry or peanut-skin-derived PACs have potent anti-inflammatory and antioxidant activities, which could alleviate the DSS-induced colitis in animal models [12,13,14]. A series of dimeric PACs such as Mahuannins A–D have been isolated from ER previously, however, the anti-UC capacity and mechanism of ER have been rarely studied [15]. In silico strategies have proven to be good tools to meet the challenges of discovering bioactive candidates from natural products with various chemical scaffolds and target profiles, as computational approaches are much more time- and labor-efficient in identifying potential active compounds and elucidating their underlying mechanisms against complex diseases [16,17].

Currently, ultra-performance liquid chromatography (UPLC) coupled with high-resolution mass spectrometry (HRMS), especially tandem mass spectrometry, is widely applied to the chemical profiling of food and plants [18,19], and new data mining skills based on mass defect filtering (MDF), diagnostic fragment ions and feature-based molecular networking have greatly facilitated the detection and identification of new compounds in foods and herbal medicines [20,21].

In the present study, UPLC coupled with linear ion trap-Orbitrap (LTQ-Orbitrap) HRMS was first employed to systematically screen PACs in ER and rapidly identify the PAC-enriched fraction. Secondly, the antioxidant and anti-inflammation activities of the ethyl acetate extract from ER was tested. Furthermore, its preventative effects on dextran-sulfate-sodium-induced ulcerative colitis in mice was investigated. Our study not only provided a strategy to rapidly screen PACs from various sources, but also laid a scientific foundation for the quality control of ER. Moreover, considering the FDA ban on dietary supplements with Ephedra alkaloids from the stems of ES, our research will greatly contribute to the full utilization of Ephedra plants.

## 2. Materials and Methods

### 2.1. Reagents and Chemicals

HPLC-grade acetonitrile and formic acid were supplied by Merck (Darmstadt, Germany) and Shanghai Acmec Biochemical Company (Shanghai, China), respectively. Analytical-grade ethanol was acquired from Greagent (Taitan, Shanghai). Ultrapure water was obtained from a Milli-Q water purification system (Millipore, MA, USA). Reference standards of Procyanidin A1, A2, B1 and B2 were purchased from TCM innovation and Standard Globalization lab of Chengdu Biopurify Phytochemicals (Chengdu, China). Fetal bovine serum (FBS) was bought from Gibco. Lipopolysaccharide (LPS) was obtained from Sigma Aldrich (Shanghai, China). Dextran sulfate sodium salt (DSS) was purchased from MP Biomedicals (Shanghai, China).

### 2.2. Plant Materials and Sample Preparation

RE was collected from Inner Mongolia in 2020 and authenticated by Prof. Haobin Hu (Jiangsu institute for food and drug control). A voucher specimen (No. G20200907) was deposited at the Medical College of Yangzhou University. The powdered sample (220 g) was accurately weighed and ultrasonically extracted twice with 2200 mL of 95% ethanol for 2 h at 60 °C. The extracts were filtered, evaporated and resuspended in water. After successive partition with n-hexane, ethyl acetate and n-butanol, the ethyl acetate fraction (ERE) was obtained for subsequent analysis [15].

The stock solutions of reference standards (Procyanidin A1, A2, B1 and B2) were prepared by dissolving 1 mg of each reference standard in 1 mL methanol. The stock solution was diluted to 10 μg/mL with methanol before instrumental analysis.

### 2.3. Potential Active Constituents: Chemical Profiling of ER and ERE by UPLC-LTQ-Orbitrap

Chromatographic separation was performed on an ACQUITY UPLC BEH C18 column (150 mm × 2.1 mm, 1.7 μm) (Waters Corporation, Milford, MA, USA) in a UPLC system ((Dionex, Thermo Fisher Scientific, Sunnyvale, CA, USA). The column temperature was set at 35 °C and the flow rate was 0.4 mL/min. The mobile phase consisted of A (0.1% formic acid in water) and B (acetonitrile), with an optimized gradient elution program as follows: 0.01−1 min, 10% B; 1–3 min, 15% B; 3–9 min, 15–40% B; 9–10 min, 40–95% B; 10–11.5 min, 95% B.

HRMS data were recorded on an LTQ-Orbitrap Elite mass spectrometer (Thermo Fisher Scientific, Germany) with an electrospray ionization (ESI) source in the negative ion mode. The spray voltage was 3.2 kV and the temperature for capillary and heater was 350 °C. The flow rate for sheath gas and auxiliary gas were 45 psi and 15 psi, respectively. The scan range was set from *m*/*z* 50 to 1000 with S-Lens RF level at 60%. The MS^2^ data were obtained under the collision energy of 35 eV.

### 2.4. Antioxidant Activity: The Free Radical Scavenging Activity of ERE

#### 2.4.1. DPPH Assay

An amount of 2.4 mg of DPPH was accurately weighed and then dissolved in 50 mL of anhydrous ethanol at a concentration of 0.1217 mmol/mL and stored at 4 °C. The sample to be tested was obtained by gradient dilution with ethanol. In the experiment, 0.4 mL of sample solution and 0.6 mL DPPH ethanol solution were mixed fully in a 1 mL EP tube in the dark for 30 min. The butylated hydroxylanisole (BHA) was used as positive control. The final concentration of ERE and BHA in the reaction system ranged from 12.5 to 400 μg/mL. Then, 200 μL was placed in a 96-well plate and the absorbance of A1 was measured at the wavelength of 517 nm. A2 represented the normal group without the test sample, and A0 represented the blank group without DPPH. The clearance rate was calculated using the formula below:

DPPH clearance% = [1 − (A1 − A0)/A2] × 100%

#### 2.4.2. ABTS Assay

ABTS solution (7 mM) and K_2_S_2_O_8_ solution (2.45 mM) were mixed in the same volume and stored in the dark at room temperature for 12 h. ABTS working solution was obtained by dilution with ethanol to yield a working solution with 0.7 absorbance at 745 nm and 30 °C. Then, 0.3 mL of the sample solution was mixed with 2.7 mL ABTS working solution at 30 °C for 6 min. Vitamin C (VC) was used as positive control. The final concentration of ERE and VC in the reaction system ranged from 12.5 to 400 μg/mL. Then, 200 μL was placed in a 96-well plate and the absorbance of A1 was measured at the wavelength of 745 nm. A2 represented the normal group, and the reaction system was 2.7 mL ABTS working solution and 0.3 mL of ethanol. The clearance rate was calculated using the formula below:

ABTS clearance% = [(A2 − A1)/A2] × 100%

### 2.5. In Vitro Studies: Effect of ERE on RAW 264.7 Cells

The Balb/c mouse macrophage cell line (RAW 264.7) was acquired from the Cell Bank of the Chinese Academy of Sciences (Shanghai, China). They were cultured in DMEM medium containing 10% FBS at 37 °C with 5% CO_2_.

#### 2.5.1. MTT Assay

MTT assay was performed to test the effects of ERE in the absence or presence of LPS (0.5 µg/mL) on the viability of RAW 264.7 cells. Cells were seeded in a 96-well plate (1 × 10^4^ cells per well) and pretreated with ERE at various concentration (5–320 µg/mL) for one hour. Subsequently, the cells were cultured with or without LPS (0.5 µg/mL) in an incubator at 37 °C for 24 h. The morphological changes of RAW 264.7 cells were observed by the inverted fluorescence microscopy. An amount of 10 µL of MTT solution and fresh medium were added to each well and incubated for 4 h. Afterwards, the solution of each well was replaced by 150 µL of dimethyl sulfoxide (DMSO) and the absorbance at 490 nm was measured by an EnSpire multimode plate reader (PerkinElmer, Shanghai, China).

#### 2.5.2. Effect of ERE on NO Production in LPS-Stimulated RAW 264.7 Cells

The RAW 264.7 cells were seeded in 96-well plates and pre-treated with ERE (5–160 µg/mL) for 1 h. Then 0.5 µg/mL LPS was added to the cells and incubated for another 24 h at 37 °C. Quantification of NO was performed according to the instructions of commercial kits (Beyotime Biotechnology, Shanghai, China).

### 2.6. In Silico Studies: Molecular Docking Study of Dimeric Proanthocyanidins and LPS

To determine the molecular simulation of Mahuannin A and B and C, Ephedrannin A and B and Proanthocyanidin A1 and A2 binding with lipopolysaccharide (LPS), the molecular docking study was conducted using AutoDock Vina 4.2.6 [22]. The chemical structures of dimeric proanthocyanidins were obtained from the Pubchem compound database (http://pubchem.ncbi.nlm.nih.gov/ (accessed on 1 September 2022)) with PubChem CID as 21,676,348 (Ephedrannin A), 25,051,177 (Ephedrannin B), 9,872,976 (Procyanidin A1) and 124,025 (Procyanidin A2). Mahuannin A, B and C were drawn using ChemDraw 20.0. (Perkin Elmer, Massachusetts, MA, USA) [8,23,24]. The compound’s 2D structure was then converted to a 3D structure in ChemBio3D 14.0 software, where it was optimized based on the energy minimization. The optimized three-dimensional structure of LPS was retrieved from the Protein Data Bank (ID: 6S8H) and a grid map (center coordinates: 110.524, 97.977 and 91.547; dimensions: 30 × 30 × 30 with a spacing of 1 Å) involving potential binding pocket was utilized based on docking results [25]. The binding pose with the lowest energy (expressed as kcal/mol) was selected as the best model for each docking test. The interaction between natural compounds and LPS was plotted by using LigPlot^+^.

### 2.7. In Vivo Studies: Effect of ERE on DSS-Induced Colitis Mice

#### 2.7.1. Induction of Colitis Mice and Experimental Grouping

Male Kunming mice (SPF grade, 6–8 weeks old, 30–35 g) were purchased from the Changzhou Cavens Experimental Animal Co. Ltd. (SCXK (SU) 2021-0013) with the Quality Certificate (No. 202207710). The Institutional Animal Care and Use Committee of Yangzhou University approved all the experiments, and all research procedures were in accordance with the China Laboratory Regulation Act (2017) under a Project License (SYXK(SU)2017-0044). All mice were housed in a temperature- (22 ± 2 °C) and humidity-controlled (50 ± 5%) environment with a 12 h light/dark cycle. They were adapted to the new environment for one week and fed with standard food and sterile water.

**Figure 2:**
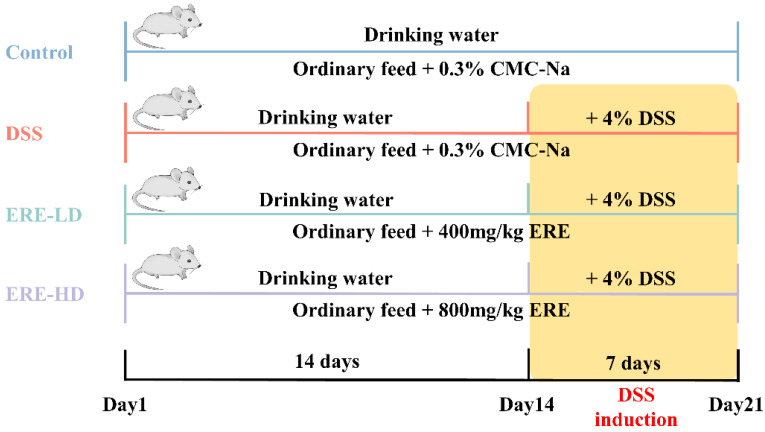
The schematic diagram of in vivo experimental design.

Twenty-four mice were randomly divided into four groups (six mice per group): the normal control group (control), the DSS model group (DSS), the DSS with a low dose of ERE (400 mg per kg body weight) group (ERE-LD) and the DSS with a high dose of ERE (800 mg per kg body weight) group (ERE-HD). The experimental design is shown in Figure 2. From day 1 to day 14, mice in all groups had free access to sterilized drinking water, and from day 15 to day 21, except for the control group, DSS was added to the drinking water at a concentration of 4% (*w*/*v*) to induce ulcerative colitis. For the control and model groups, 0.3% CMC-Na was used as the blank treatment. Mice in the ERE-LD and ERE-HD groups were given corresponding doses of ERE (dissolved in 0.3% CMC-Na). The dosage of extract and DSS were chosen based on preliminary experiment results and previous studies [26].

#### 2.7.2. Evaluation of Disease Activity Index (DAI), Colon Length and Spleen Index

During the DSS treatment period, body weight, stool characteristics and presence of blood in the stool were recorded daily and graded on a scale from 0 to 4: body weight loss (0, 0–1%; 1, 1–5%; 2: 5–10%; 3, 10–15%; 4, >15%); stool consistency (0, normal; 2, loose stools; 4, diarrhea); occurrence of gross blood in the stool (0, normal; 2, presence of blood; 4, gross bleeding). The DAI was calculated as an average score of the above-mentioned parameters. At the end of the experiment, the animals were sacrificed, and the spleen was excised and weighed. The spleen index was calculated as the spleen weight (mg) divided by the corresponding body weight (g). The colon was dissected out and the distance from the cecal–colon junction to the proximal rectum was measured.

#### 2.7.3. Histopathological Analysis

The colon samples were fixed in 4% paraformaldehyde solution and embedded in paraffin wax. The sections were stained with hematoxylin and eosin (H&E) and scored for inflammation, mucosal damage, crypt damage and range of lesions on a five-grade scale [27].

#### 2.7.4. Determination of Inflammatory Cytokines and Biochemical Assays

The colon tissues were rinsed with ice-cold PBS, accurately weighed and homogenized with PBS (*w*/*v*, 1:9) on ice. The homogenates were centrifuged at 5000× *g* for 15 min at 4 °C and the obtained supernatants were used to determine TNF-α, IL-6 and IL-10 using mouse-specific enzyme-linked immunosorbent assay (ELISA) kits (ExCell Biology, Shanghai, China) according to the manufacturer’s instructions. The protein content was quantified by the bicinchoninic acid (BCA) protein assay kit (Beyotime Biotechnology, Shanghai, China) and the results were presented as pg/mg protein of colon samples. In addition, the changes in myeloperoxidase (MPO) activity, superoxide dismutase (SOD) activity, malondialdehyde (MDA) and nitrogen monoxide (NO) in the colon homogenates were assessed by the corresponding commercial kits (Nanjing Jiancheng Co., Ltd., Nanjing, China).

### 2.8. Statistical Analysis

GraphPad Prism 8.0 (GraphPad software, Inc., Beijing, China) and SPSS 25.0 (SPSS Inc., Chicago, IL, USA) were used to analyze the animal experiment data. Student’s *t*-test or one-way analysis of variance were used to determine the statistical significance. The data were expressed as mean ± standard deviation and differences between groups were considered significant at *p* < 0.05.

## 3. Results

### 3.1. The Integrated Strategy based on Neutral Loss Filtering and Diagnostic Fragmentation Pattern to Identify Potential PACs

The building blocks of PACs are flavan-3-ols, mainly including (epi)afzelechin, (epi)catechin and (epi)gallocatechin (Figure 1). Structures of PACs are diversified because of the degree of polymerization, the interflavan linkage and the type of flavan-3-ols (with substitutions) [28]. It has been reported that the PAC dimer composed of a top part (extension unit, T) and a bottom part (terminal unit, B) has three main fragmentation pathways, quinone methide fission (QM), heterocyclic ring fission (HRF) and Retro-Diels-Alder fission (RDA), which was also confirmed in our study using Procyanidin A1, Procyanidin A2, Procyanidin B1 and Procyanidin B2 as reference standards. The product ion spectra of Procyanidin A1, Procyanidin A2, B1 and B2 are shown in Figure S1, and the proposed fragmentation route of Procyanidin A2 and Procyanidin B2 is shown in Figure 1. Procyanidin A2 consists of two (-)-epicatechin units linked through one interflavan carbon bond (C4-C8) and one ether bond (C2-O-C7), while Procyanidin B2 is composed of two molecules of (-)-epicatechin solely connected by one C4-C8 bond, therefore the [M-H]^−^ of A-type dimeric PACs is 2 Da less than the B-type linkage. In addition, the linkage sequence can be inferred through QM cleavage of the top (T) and bottom (B) parts, as the A-type dimer Procyanidin A2 could generate QM product ions of *m*/*z* 289.0718 [MB-H]^−^ and *m*/*z* 285.0411 [MT-5H]^−^ and the B-type dimer Procyanidin B2 would produce QM product ions of *m*/*z* 289.0718 [MB-H]^−^ and *m*/*z* 287.0565 [MT-3H]^−^. The RDA product ions of *m*/*z* 423.0728 for Procyanidin A2 and *m*/*z* 425.0878 for Procyanidin B2 all indicated a loss of 152 Da, suggesting that the B ring has two hydroxyl groups but the RDA fragmentation mainly occurs at the top and bottom unit in A-type and B-type dimers, respectively. Additionally, a loss of 126 Da in HRF fragmentation resulted in *m*/*z* 449.0886 for Procyanidin A2 and *m*/*z* 451.1036 for Procyanidin B2, which indicates a 1,3,5-trihydroxybenzene structure in the A ring. Diagnostic fragmentation ions derived from QM, RDA and HRF fission could only provide references for the linkage type and sequence. Procyanidin B1 consisting of epicatechin and catechin units could not be differentiated from Procyanidin B2 by product ion spectra (Figure S1), as compounds with differences only in linkage position (C4-C8 or C4-C6) or optical structure of monomeric units share almost the same fragmentation patterns [29]. However, some isomers can be chromatographically separated because of structural differences, which makes UPLC ideal for rapid screening of PACs [30].

### 3.2. Potential Active Constituents in ERE and the Antioxidant Activity

**Figure 3:**
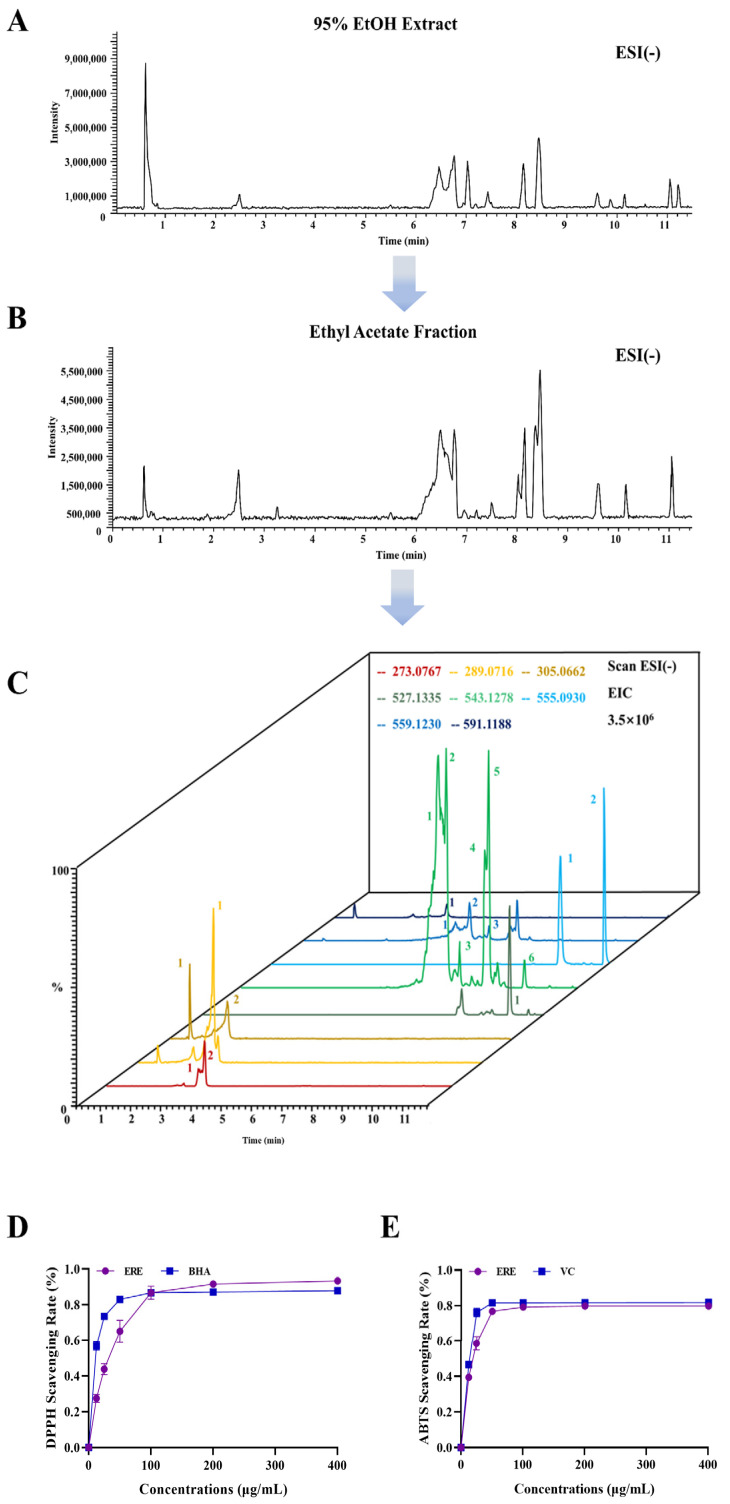
The total ion chromatogram (TIC) of 95% ethanol extract (**A**) and the ethyl acetate fraction (**B**) and the extract ion chromatogram (EIC) of the ethyl acetate fraction (**C**) in the negative mode. The DPPH (**D**) and ABTS (**E**) scavenging rate of the ethyl acetate fraction. (BHA: butylated hydroxylanisole, positive control; VC: vitamin C, positive control).

In the present study, UPLC-LTQ-Orbitrap-HRMS was applied to chemical profiling of dimeric PACs in RE, which generated a relatively large amount of untargeted MS and MS/MS data. The total ion chromatogram (TIC) of 95% ethanol extract (A) and the ethyl acetate fraction (B) and the extract ion chromatogram (EIC) of the ethyl acetate fraction (C) in the negative mode are presented in Figure 3. The potential precursor ions for PPs, PCs and PDs in RE were searched, and then the precursor-to-product ion transition information were extracted. After screening with the diagnostic ions verified by product ion spectra of standard compounds, potential dimeric PACs in RE could be tentatively identified (Table 1). For the proanthocyanidin dimers, we can determine the linkage type through the accurate quasi-molecular ion [M-H]^−^, and product ions originated from RDA, QM and HRF fragmentation could only be used to determine the connection sequence of (epi)afzelechin, (epi)catechin and (epi)gallocatechin units, but not to distinguish between epimers [29,30]. The DPPH assay showed that the IC50 values for ERE and BHA were 30.30 and 10.17 μg/mL. The ABTS assay revealed that the IC50 values for ERE and VC were 18.00 and 13.16 μg/mL. They collectively demonstrated that ERE had good antioxidant capabilities [31,32].

### 3.3. Effects of ERE on Viability and NO Production of LPS-Stimulated RAW 264.7 Cells

**Figure 4:**
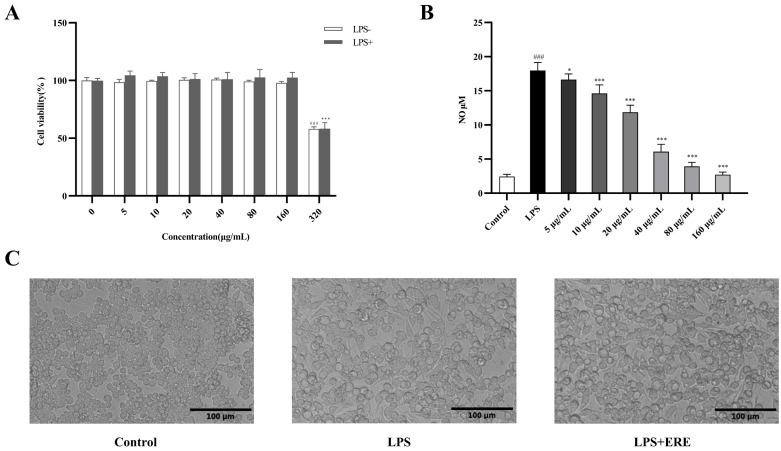
The effects of ERE on cell viability (**A**), NO production (**B**) and cell morphology (**C**) in LPS-induced RAW 264.7 cells. Scale bar = 100 μm. The values are expressed as the mean ± S.D: * *p* < 0.05 compared with LPS group; *** *p* < 0.001 compared with LPS group; ### *p* < 0.001 compared with control group.

The effects of ERE on viability of RAW 264.7 cells in the absence or presence of LPS are presented in Figure 4A, and ERE at the concentration of 320 µg/mL (*p* < 0.001) significantly inhibited the cell viability with or without LPS stimulation. Therefore, ERE in the range of 5 to 160 µg/mL was used in the following experiments. Additionally, morphology changes were observed in RAW 264.7 cells and LPS-stimulated RAW 264.7 cells before and after ERE (160 µg/mL) treatment. As shown in Figure 4C, LPS stimulation induced changes in cellular morphology, indicating the successful induction of the inflammation model. Meanwhile, pretreatment with ERE suppressed the LPS-induced cell morphology changes.

The effect of ERE on LPS-induced NO release in RAW 264.7 cells is shown in Figure 4B. When cells were stimulated by LPS, NO production was increased significantly, and ERE could dose-dependently inhibit the release of NO. The inhibition rates were 7.4%, 18.64%, 34.00%, 66.07%, 78.13% and 84.83% at 5, 10, 20, 40, 80 and 160 µg/mL concentrations of ERE, respectively.

### 3.4. Molecular Docking Study of Dimeric Proanthocyanidins and LPS

**Figure 5:**
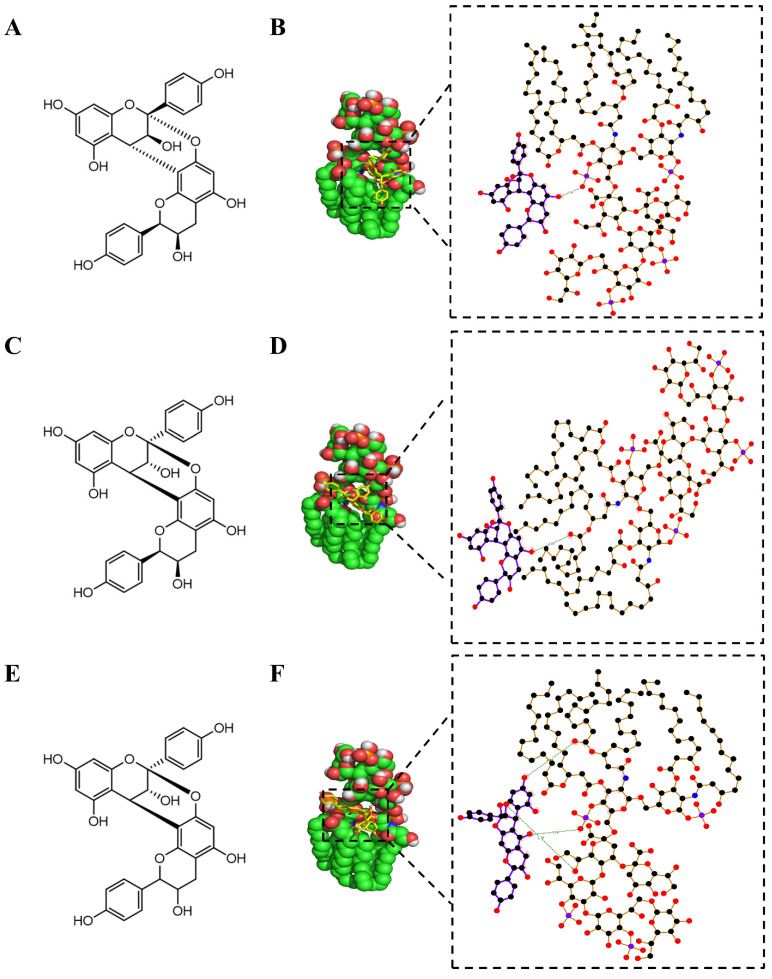
Computer modeling of the LPS and selected natural compounds binding. (**A**) Two-dimensional structure of Mahuannin A. (**B**) The preferred orientation of Mahuannin A in complex with LPS. (**C**) Two-dimensional structure of Mahuannin B. (**D**) The preferred orientation of Mahuannin B in complex with LPS. (**E**) Two-dimensional structure of Mahuannin C. (**F**) The preferred orientation of Mahuannin C in complex with LPS (black circles mean carbon atoms; red circles mean oxygen atoms; blue circles mean nitrogen atoms; purple circles mean phosphorus atoms; the green dashed line means hydrogen bond; 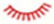 means hydrophobic contact; 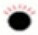 means atoms involved in hydrophobic contact).

Seven dimeric A-type PACs exhibited good binding character with LPS, as shown in Table S1, demonstrating binding affinity equal to or less than −7.0 kcal/mol. As shown in Figure 5, phenolic hydroxyl groups of Mahuannin A were involved in the formation of hydrogen bonds between the phosphate groups of LPS and Mahuannin A, while the carbonyl groups of Mahuannin C were involved in hydrogen bonding between fatty acyl chains of LPS and Mahuannin C. The carbonyl groups of Mahuannin B also formed hydrophobic interactions with the fatty acyl chains of LPS. Additionally, the benzene rings of both Ephedrannin A and Ephedrannin B formed covalent bonds with phosphate groups of LPS, which exhibited strong interaction among selected natural compounds in this study (Figure S2). As seen from the conformation of the preferred orientation, Procyanidin A2 was in close contact with the 3-deoxy-D-manno-octulosonate (KDO) moiety of LPS to form hydrogen bonding, while the carbonyl groups of Procyanidin A1 formed hydrophobic interactions with the fatty acyl chains of LPS (Figure S3).

### 3.5. ERE Relieved DSS-Induced Colitis

**Figure 6:**
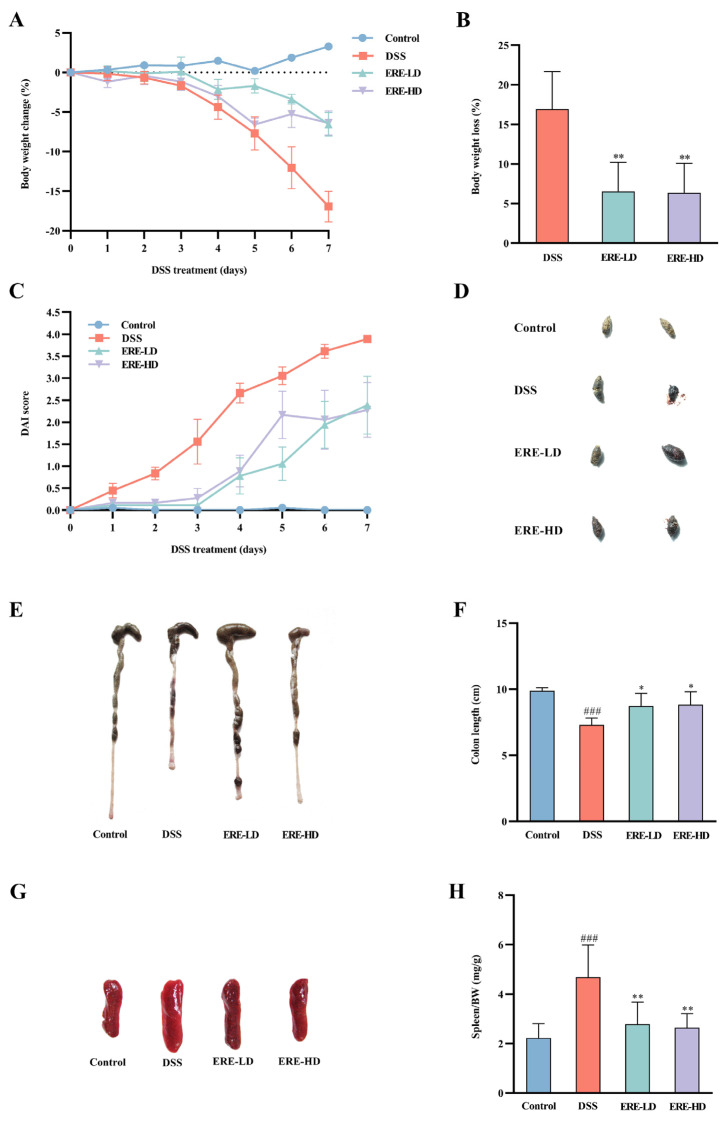
Effects of ERE on DSS-induced UC mice. The percentage of body weight change during DSS treatment (**A**). The total weight loss relative to the baseline value at day 0 (**B**). Disease activity index (DAI) scores calculated during DSS treatment (**C**). Representative photographs of feces before and after DSS treatment (**D**). Representative photos of colons € and the colon length in different groups (**F**). Representative photos of spleens (**G**) and the spleen/BW ratios in different groups (**H**). The values are expressed as the mean ± S.D.: * *p* < 0.05 compared with DSS group; ** *p* < 0.01 compared with DSS group; ### *p* < 0.001 compared with control group.

During DSS intervention, the mice body weight and DAI scores were recorded daily. As shown in Figure 6A, the mice in the DSS model group suffered from a decrease in body weight after day 3. At the end of the experiment, DSS treatment induced 16.93% weight loss compared with the baseline value; however, the mice in the ERE group experienced a significant improvement (*p* < 0.01) in weight loss (Figure 6B). DAI scores (Figure 6C) were used to evaluate the body weight loss, stool characteristics and presence of blood. The DSS model group was dramatically increased after DSS induction compared with the normal control group; however, the DAI scores were significantly lower in the ERE-LD and ERE-HD groups compared with the DSS model group. Representative photos of feces from different groups are shown in Figure 6D. In the DSS-induced colitis model, shortening of colon length has been considered as a marker for disease severity [33]. The colons of mice in the DSS model group were much shorter than those in the control group (*p* < 0.001) (Figure 6E,F). Nonetheless, ERE treatment exerted preventative effects on the colon length of DSS-induced colitis (*p* < 0.05) (Figure 6E,F). In addition, the spleen index in the DSS model group was markedly increased compared with the normal control group (*p* < 0.001), but ERE treatment could greatly reverse the trend (*p* < 0.01).

### 3.6. ERE Ameliorated Intestinal Damage

**Figure 7:**
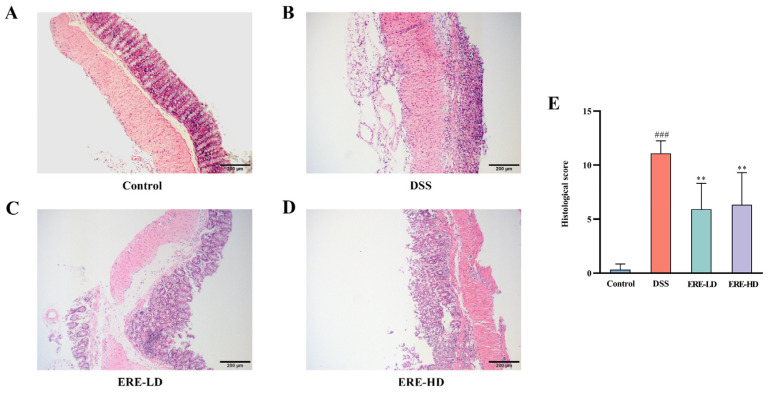
Effects of ERE on the histopathological changes in the colon tissues. Representative H&E staining sections of colon tissues (**A**–**D**). Scale bar = 200 μm. (**E**) Histopathology scores of colon tissues in different groups. The values are expressed as the mean ± S.D.: ** *p* < 0.01 compared with DSS group; ### *p* < 0.001 compared with control group.

To further evaluate the impact of ERE on the colon, histopathology examinations were performed. Representative photos of colon sections after H&E staining are shown in Figure 7. According to Figure 7A, the colon tissues in the control group were highly structured and exhibited compact columnar epithelium and intact intestinal crypts, with no histological abnormalities. On the contrary, the colons in the DSS model group displayed epithelial destruction, mucosal damage, crypt disappearance and inflammatory cell infiltration in the submucosa and muscular layer. However, after treatment with ERE, these pathological alterations induced by DSS were greatly improved. Additionally, the histological score for each mouse was given blindly by the pathologist according to the five-grade scale mentioned previously. As shown in Figure 7E, the DSS model group (11.08 ± 1.16) had the highest score among all groups, and the score was significantly higher than the control group (*p* < 0.001). However, the histological scores of the ERE-LD (5.92 ± 2.40) and ERE-HD (6.33 ± 2.98) groups were both significantly lower than the DSS model group (*p* < 0.01), indicating that ERE exerted a protective effect in preventing DSS-induced colitis.

### 3.7. ERE Exerted Intestinal Anti-Inflammatory Effects

**Figure 8:**
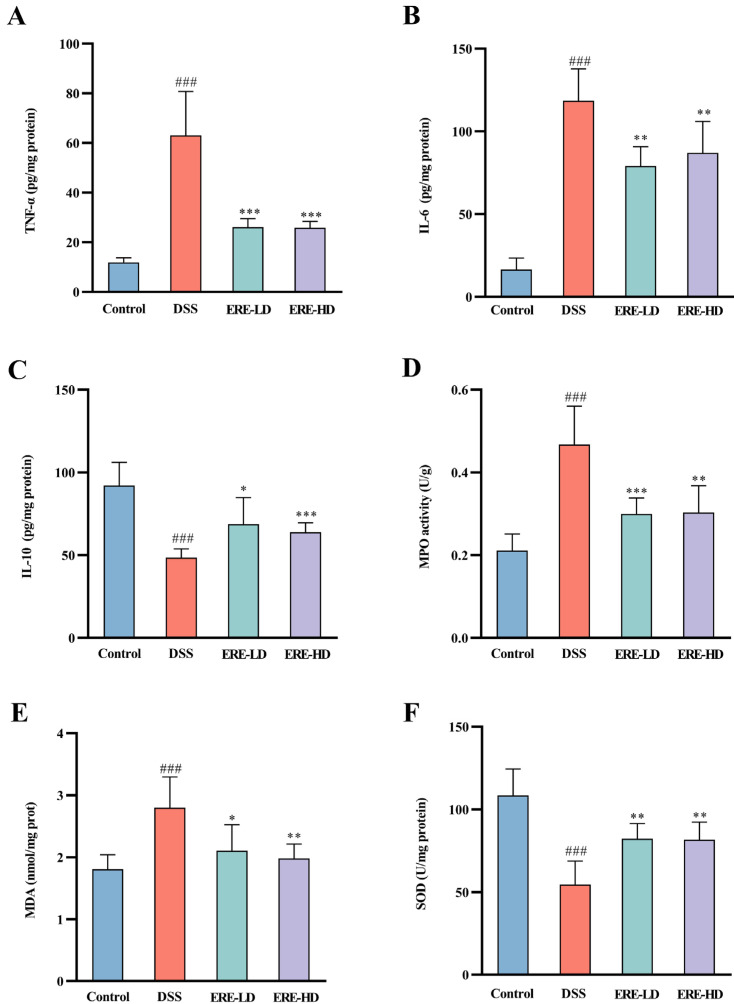
Effects of ERE on inflammatory cytokines and the activity of enzymes in the colon. The cytokine levels of TNF-α, IL-6 and IL-10 among different groups (**A**–**C**). The myeloperoxidase (MPO) activity (**D**), malondialdehyde (MDA) levels (**E**) and superoxide dismutase (SOD) activity (**F**) in the colon tissues from different groups. The values are expressed as the mean ± S.D: * *p* < 0.05 compared with DSS group; ** *p* < 0.01 compared with DSS group; *** *p* < 0.001 compared with DSS group; ### *p* < 0.001 compared with control group.

To evaluate the potential anti-inflammatory effects of ERE, inflammatory cytokines such as TNF-α, IL-6 and IL-10 were analyzed in colon tissues. The TNF-α (*p* < 0.001) and IL-6 (*p* < 0.01) levels in the DSS model group were significantly elevated compared with the control group, and ERE treatment significantly reduced the concentration of TNF-α (*p* < 0.001) and IL-6 (*p* < 0.01) in colon tissue (Figure 8A,B). Additionally, a decrease in the concentration of IL-10 was found in the DSS group (48.58 ± 5.26); however, ERE treatment could significantly upregulate the IL-10 level (Figure 8C, *p* < 0.05).

### 3.8. ERE Suppressed MPO Activity in Colon Tissues

As shown in Figure 8D, the MPO activity in colon tissues from DSS model group (0.47 ± 0.09) were markedly increased compared with that from the control group (*p* < 0.001). In contrast, the elevated MPO activities were significantly suppressed after ERE treatment (*p* < 0.01).

### 3.9. ERE Regulated Oxidative Stress

To evaluate the effects of ERE on oxidative stress in DSS-induced ulcerative colitis, MDA level and SOD activity of the colon were measured. According to Figure 8E,F, mice in the DSS group showed a marked upregulation in MDA level (2.80 ± 0.49) accompanied by a significant reduction in SOD activity (54.68 ± 14.1); however, ERE treatment could significantly decrease the MDA level and increase the SOD activity in the DSS-induced colon tissues (*p* < 0.05).

## 4. Discussion

Proanthocyanidins (PACs), also known as condensed tannins, are popular health-promoting compounds synthesized in plants to defend against stress conditions such as various pathogen infections [34]. PACs can be classified into three categories according to the type of flavan-3-ol unit. PACs exclusively composed of (epi)catechin are called Procyanidins (PCs), and PACs containing at least one (epi)afzelechin or (epi)gallocatechin are designated as propelargonidins (PPs) and prodelphinidins (PDs), respectively. Our previous untargeted plant metabolomics approach to study the chemical difference of the stems and roots of ES revealed that they contained different types of alkaloids and flavonoids. Combined with results from the absolute quantification of ephedrine and pseudoephedrine, it has been shown that the stems of ES were rich in ephedrine-type alkaloids and the roots were rich in macrocyclic spermine alkaloids [7]. In the present study, we found that dimeric PACs derived from ERE not only contained (epi)catechin, (epi)afzelechin and (epi)gallocatechin, but also involved apigeniflavan and kaempferol. Among them, the content of A-type dimeric PACs made up of (epi)afzelechins was relatively higher, which may contribute to the good antioxidant activities determined by DPPH and ABTS assays.

To evaluate the in vitro anti-inflammation activity of ERE, the mouse macrophage cell RAW264.7 was used [35]. When stimulated by LPS, macrophages, as the most responsive immune sensors and reactors, generated pro-inflammatory cytokines and NO to boost the inflammatory response [36,37]. Our results showed that pretreatment with ERE could inhibit the release of LPS-induced NO production. Considering the relatively higher levels of A-type PACs in ERE, the molecular interaction between dimeric A-type PACs and LPS was performed. Procyanidin A1 and A2 are the most commonly reported dimeric A-type PACs, which have been found in fruits and beverages. Ephedrannin A, Ephedrannin B, as well as Mahuannin A, B and C are dimeric A-type PACs that have been reported in the roots of *Ephedra sinica*. Therefore, the seven compounds were selected to study whether dimeric A-type PACs interacted with LPS by binding directly.

In our study, the docking characteristics of Ephedrannin A and B and Mahuannin A, B and C with LPS were first explored and compared with Procyanidin A1 and A2. Zheng at al. performed molecular docking analysis of Procyanidin B2 binding to LPS and found that Procyanidin B2 and LPS showed favorable hydrogen bonding and hydrophobic interactions, which was further validated by the experimental result that Procyanidin B2 could dose-dependently inhibit the LPS-induced release of TNF-α from RAW 264.7 cells [25]. The phenolic hydroxyl groups of Procyanidin B2 and the fatty acyl chains of LPS formed hydrogen bonds, and the carbonyl groups also contributed to the hydrogen bonding between the KCO moiety of LPS and Procyanidin B2. Additionally, the hydrophobic interactions involved the benzene rings of Procyanidin B2 and fatty acyl chains of LPS. In our previous chemical profiling study, the building blocks and linkage types of dimeric PACs derived from ERE were different from Procyanidin B2, and our molecular simulation results revealed that Procyanidin A1 and A2 and dimeric PACs derived from ERE, especially Mahuannin A, B and C, exhibited better binding affinity with LPS. Altogether, the binding of PACs to LPS is quite different from that of polymyxin B (PMB), a well-known LPS-binding agent that usually possesses positive charges to attract the phosphate groups of LPS [38]. It has been reported that PACs could have anti-inflammatory activities via inhibition of the mRNA expressions of COX-2, iNOS enzymes, and IL-1β, IL-6 and TNF-α cytokines [39]. It is expected that dimeric A-type PACs screened out from ERE may become novel lead compounds against LPS-induced inflammatory responses and would also be developed as immune response antagonists against various inflammatory diseases.

Accordingly, the in vivo anti-inflammatory effects of ERE were tested. UC is an inflammatory bowel disease with a high prevalence in developed countries and an increasing incidence in developing countries. Current therapies could alleviate the symptoms of UC, but most have undesirable side effects. Complementary therapies including the use of plant-derived phytochemicals have been investigated, and polyphenols such as proanthocyanidins showed promising results [40]. In order to reveal the pathological mechanism of UC, trinitrobenzene sulfonic acid, oxazolone and DSS are the most commonly used chemicals to induce colitis in experimental murine models, and the DSS-induced mice model could cause epithelial mucosa damage that is more similar to UC [41].

In the present study, except for the obvious UC symptoms, we observed an increase in pro-inflammatory cytokines (TNF-α, IL-6) and MPO activity as well as a decrease in anti-inflammatory cytokines such as IL-10, which are closely related to the severity of colitis [42]. Cytokines secreted form immune cells such as macrophages, T cells and dendritic cells play a crucial role in UC pathogenesis [43]. When UC occurs, the inflammatory cells in the body are activated to produce a series of inflammatory reactions. Neutrophil infiltration is one of the markers of colonic mucosal inflammation. MPO is rich in neutrophils, and its level changes reflect the active state of neutrophils. Therefore, MPO is often used as an indicator to evaluate the degree of colonic inflammation in experimental studies [44]. Additionally, oxidative stress is another hallmark of UC, as the increased activity of phagocytic leukocytes in the colons of UC patients can lead to enhanced production of pro-oxidant molecules [45]. MDA is the product of free radical lipid peroxidation in the body, and its expression level is positively correlated with tissue oxidative stress injury [46]. SOD is an antioxidant enzyme existing in organisms, which has the effects of scavenging oxygen free radicals and inhibiting lipid peroxidation. When the level of oxygen free radicals exceeds the body’s ability to remove them, SOD is exhausted, leading to the upregulated MDA level in the colon tissue [47,48].

Our results demonstrated that ERE effectively attenuated DSS-induced colitis in mice through inhibiting inflammation and oxidative stress. ERE contained mainly PAC monomers and dimers, which exhibited higher absorption rates than trimers or tetramers, and the bioavailability and pharmacological activity is heavily dependent on its degree of polymerization [49]. Compared with B-type PACs, the number of reports on the pharmacological study of A-type AC is much smaller. Huang et al. revealed the mechanism of A-type Procyanidins from peanut skin (PSP) on DSS-induced colitis in Balb/c mice and showed that PSP administration could regulate the expression of inflammatory cytokines and oxidative stress (MDA, T-SOD, NO and iNOS) in mice, as well as gut microbiota and metabolism. Kitabatake et al. found that persimmon (Ebenaceae Diospyros kaki Thunb.)-derived tannins consisting of catechin groups could relieve the DSS-induced colitis in female mice through suppression of the inflammatory response and alteration of microbiota composition [50]. The composition of A-type PACs is slightly different from PSP and persimmon-derived tannins, as dimers consisting of (epi)afzelechins constitute a large proportion in ERE. As far as we know, this is the first study to explore the preventative effects of ERE on DSS-induced colitis in mice. Considering the potential gastrointestinal health of dietary PACs and their interactions with gut microbiota, the effects of ERE on gut microbiota deserve further study [51]. Additionally, Wu et al. discovered the chemopreventive effects of whole cranberry powder (WCP) on colitis-associated mouse colon tumorigenesis induced by azoxymethane (AOM) and dextran sulfate sodium (DSS), which provided new insights into the therapeutic effects of PACs on promoting colon health [52].

## 5. Conclusions

In the present study, UPLC-LTQ-Orbitrap-MS was employed to systematically analyze the chemical profile of RE ethanolic and ethyl acetate extract for the first time. A total of 20 proanthocyanidin monomers and dimers were rapidly screened out and they were enriched in the ethyl acetate extract from the roots of *Ephedra Sinica*. We also demonstrated that ethyl acetate extract could not only inhibit the NO production in LPS-induced RAW 264.7 cells but also had preventative effects on dextran-sulfate-sodium-induced ulcerative colitis in mice through inhibiting inflammatory cytokines and regulating oxidative stress. These results laid a scientific foundation for better quality control and pharmacological studies of RE and for future studies on its bioactivity. Furthermore, our findings provide empirical evidence for the potential health-promoting benefits of ethyl acetate extract from RE as a good source for A-type proanthocyanidins.

## Figures and Tables

**Table 1:** The tentative identification of chemical compounds in the ERE.

No.	t_R_ (min)	M.F.	[M-H]^−^ *m*/*z*	Fragment Ions	Tentative Identification (Connection Sequence for PAC Dimers)
273-1	3.04	C_15_H_14_O_5_	273.0767	229.0878, 205.0870, 189.0565, 187.0766, 161.0612, 147.0459, 137.0249, 135.0457, 123.0460	(Epi)afzelechin
273-2	3.25	C_15_H_14_O_5_	273.0768	229.0864, 205.0871, 189.0560, 187.0776, 161.0608, 147.0455, 137.0249, 135.0453, 123.0453	(Epi)afzelechin
289-1	2.49	C_15_H_14_O_6_	289.0716	245.0821, 221.0823, 205.0509, 195.0302, 151.0405, 149.0248, 137.0249, 123.0456, 109.0299	Catechin
305-1	0.66	C_15_H_14_O_7_	305.0663	287.0561, 269.0457, 179.0352, 137.0249, 125.0249, 109.0299	(Epi)gallocatechin
305-2	1.87	C_15_H_14_O_7_	305.0662	287.0561, 269.0461, 179.0353, 137.0249, 125.0249, 109.0299	(Epi)gallocatechin
527-1	10.15	C_30_H_24_O_9_	527.1335	401.1150, 391.0930, 379.0910, 323.0941, 307.0618, 273.0771, 253.0509, 229.0873, 125.0249	Apigeniflavan-A-(epi)afzelechin
543-1	6.49	C_30_H_24_O_10_	543.1281	417.0970, 407.0764, 273.0771, 269.0459, 248.9584, 212.0738, 174.9561, 146.9662, 112.9859	(Epi)afzelechin-A-(epi)afzelechin
543-2	6.76	C_30_H_24_O_10_	543.1279	417.0937, 407.0727, 273.0739, 269.0430, 212.0732, 174.9552, 146.9661, 112.9858, 96.9604	(Epi)afzelechin-A-(epi)afzelechin
543-3	7.19	C_30_H_24_O_10_	543.1281	417.0981, 297.0771, 273.0772, 269.0461, 212.0749, 146.9662, 112.9859, 96.9603	(Epi)afzelechin-A-(epi)afzelechin
543-4	8.02	C_30_H_24_O_10_	543.1278	417.1088, 407.0768, 273.0768, 269.0456, 212.0748, 174.9560, 146.9661, 129.9760, 112.9858	(Epi)afzelechin-A-(epi)afzelechin
543-5	8.15	C_30_H_24_O_10_	543.1279	417.1123, 407.0902, 273.0772, 269.0458, 212.0736, 174.9562, 146.9662, 112.9858, 96.9603	(Epi)afzelechin-A-(epi)afzelechin
543-6	9.32	C_30_H_24_O_10_	543.1281	417.0975, 407.0769, 289.0716, 253.0506, 245.0820, 212.0749, 179.0353, 112.9858, 96.9603	Apigeniflavan-A-(epi)catechin
555-1	9.56	C_30_H_20_O_11_	555.0919	469.1173, 441.1173, 349.0720, 333.0771, 291.0664, 285.0405, 269.0457, 149.0249	(Epi)afzelechin-A-kaempferol
555-2	11.04	C_30_H_20_O_11_	555.0930	469.1157, 441.1154, 349.0714, 333.0772, 291.0664, 285.0403, 269.0456, 149.0248	(Epi)afzelechin-A-kaempferol
559-1	4.95	C_30_H_24_O_11_	559.1233	523.1354, 433.1074, 405.1084, 317.0663, 289.0718, 269.0454, 245.0823	(Epi)afzelechin-A-(epi)catechin
559-2	5.50	C_30_H_24_O_11_	559.1230	523.1396, 433.1106, 407.0908, 289.0717, 269.0457, 245.0827	(Epi)afzelechin-A-(epi)catechin
559-3	6.16	C_30_H_24_O_11_	559.1231	523.1398, 455.1356, 433.1115, 407.0914, 289.0716, 269.0457, 245.0820	(Epi)afzelechin-A-(epi)catechin
591-1	3.72	C_30_H_24_O_13_	591.1127	555.1358, 465.1054, 407.0904, 301.0355, 289.0720, 175.0042, 125.0250	(Epi)gallocatechin-A-(epi)catechin

(Epi)afzelechin→afzelechin or epiafzelechin; (epi)catechin→catechin or epicatechin; (epi)gallocatechin→gallocatechin or epigallocatechin; A→A-type proanthocyanidin (for proanthocyanidin dimers, the monomeric unit on the left side represents the top part and epimers in the dimer cannot be distinguished).

## Data Availability

The data presented in this study are available in article and Supplementary Materials.

## Supplementary Materials

The following supporting information can be downloaded at: https://www.mdpi.com/article/10.3390/metabo12100957/s1, Table S1: The binding affinity between Mahuannin A and B and C, Ephedrannin A and B and Procyanidin A1 and A2 with lipopolysaccharide (LPS). Figure S1: MS/MS spectra of Procyanidin A1 (Rt 3.34 min), A2 (Rt 4.63 min), B1 (Rt 1.39 min) and B2 (Rt 2.16 min). Figure S2: Computer modeling of the LPS and selected natural compounds binding. (A) Two-dimensional structure of Ephedrannin A; (B) the preferred orientation of Ephedrannin A in complex with LPS; (C) two-dimensional structure of Ephedrannin B; (D) the preferred orientation of Ephedrannin B in complex with LPS (black circles mean carbon atoms; red circles mean oxygen atoms; blue circles mean nitrogen atoms; purple circles mean phosphorus atoms; purple lines mean covalent bonds; red dashed lines mean π-stacking interaction;  means hydrophobic contact;  means atoms involved in hydrophobic contact). Figure S3: Computer modeling of the LPS and selected natural compounds binding. (A) Two-dimensional structure of Procyanidin A1; (B) the preferred orientation of Procyanidin A1 in complex with LPS; (C) two-dimensional structure of Procyanidin A2; (D) the preferred orientation of Procyanidin A1 in complex with LPS (black circles mean carbon atoms; red circles mean oxygen atoms; blue circles mean nitrogen atoms; purple circles mean phosphorus atoms; green dashed lines mean hydrogen bonds;  means hydrophobic contact;  means atoms involved in hydrophobic contact).
