# Supplementary material for: Rapid Screening of Proanthocyanidins from the Roots of Ephedra sinica Stapf and its Preventative Effects on Dextran-Sulfate-Sodium-Induced Ulcerative Colitis
Source: Metabolites. 2022 Oct 10;12(10):957. doi: 10.3390/metabo12100957 (PMC9611049; doi:10.3390/metabo12100957)
Supplement: Supplementary file 1 [file metabolites-12-00957-s001.zip › metabolites-1941973-supplementary.pdf]

metabolites

MDPI

Supplementary Materials

Table S1. The binding affinity between Mahuannin A & B & C, Ephedrannin A & B, and Procyanidin A1 & A2 with lipopolysaccharide (LPS).

Compounds	Binding affinity (kcal/mol)
Mahuannin A	-8.0
Mahuannin B	-9.3
Mahuannin C	-7.9
Ephedrannin A	-7.3
Ephedrannin B	-7.6
Procyanidin A1	-7.5
Procyanidin A2	-7.2

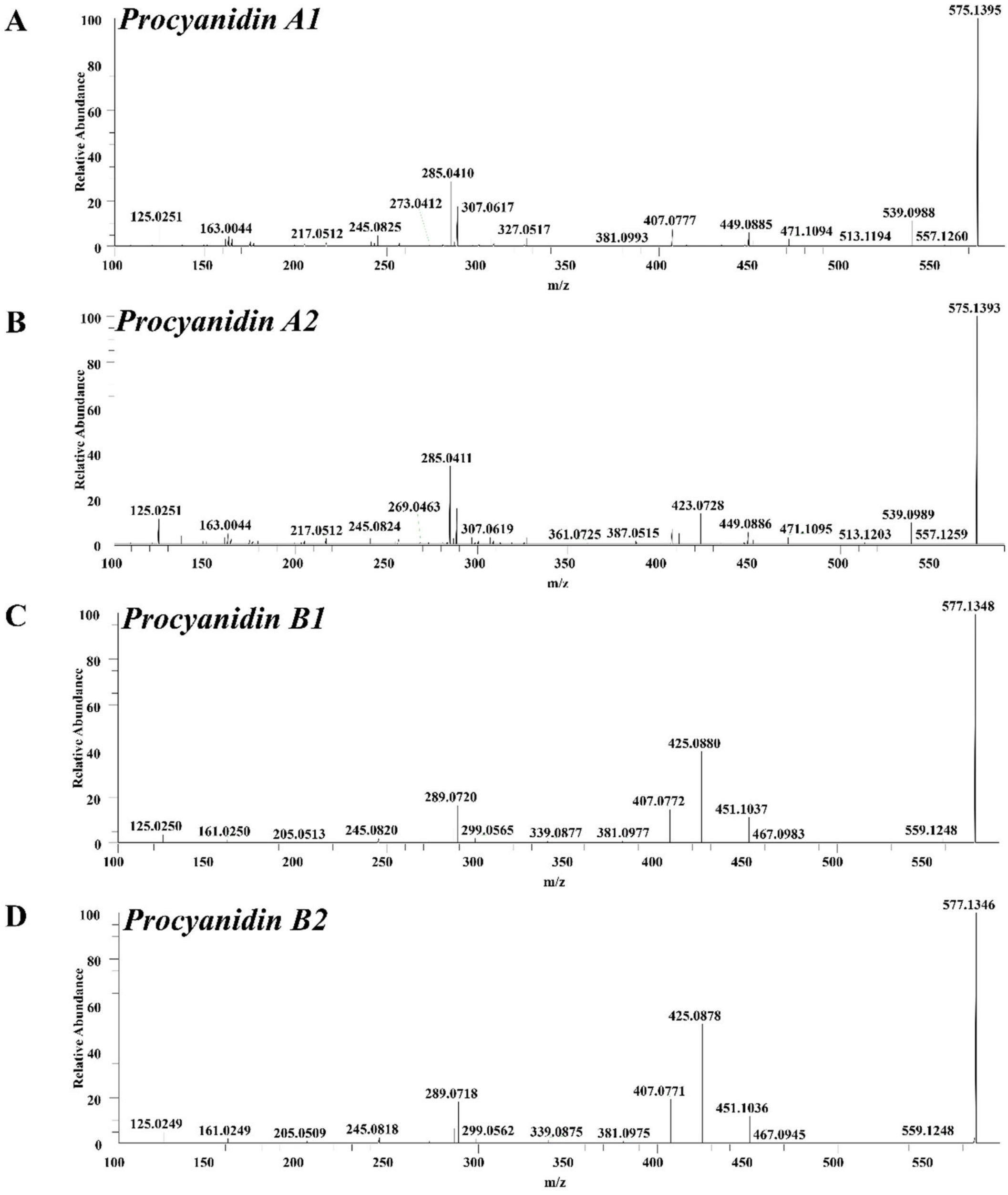

Figure S1. MS/MS spectra of Procyanidin A1 (Rt 3.34 min), A2 (Rt 4.63 min), B1 (Rt 1.39 min) and B2 (Rt 2.16 min).

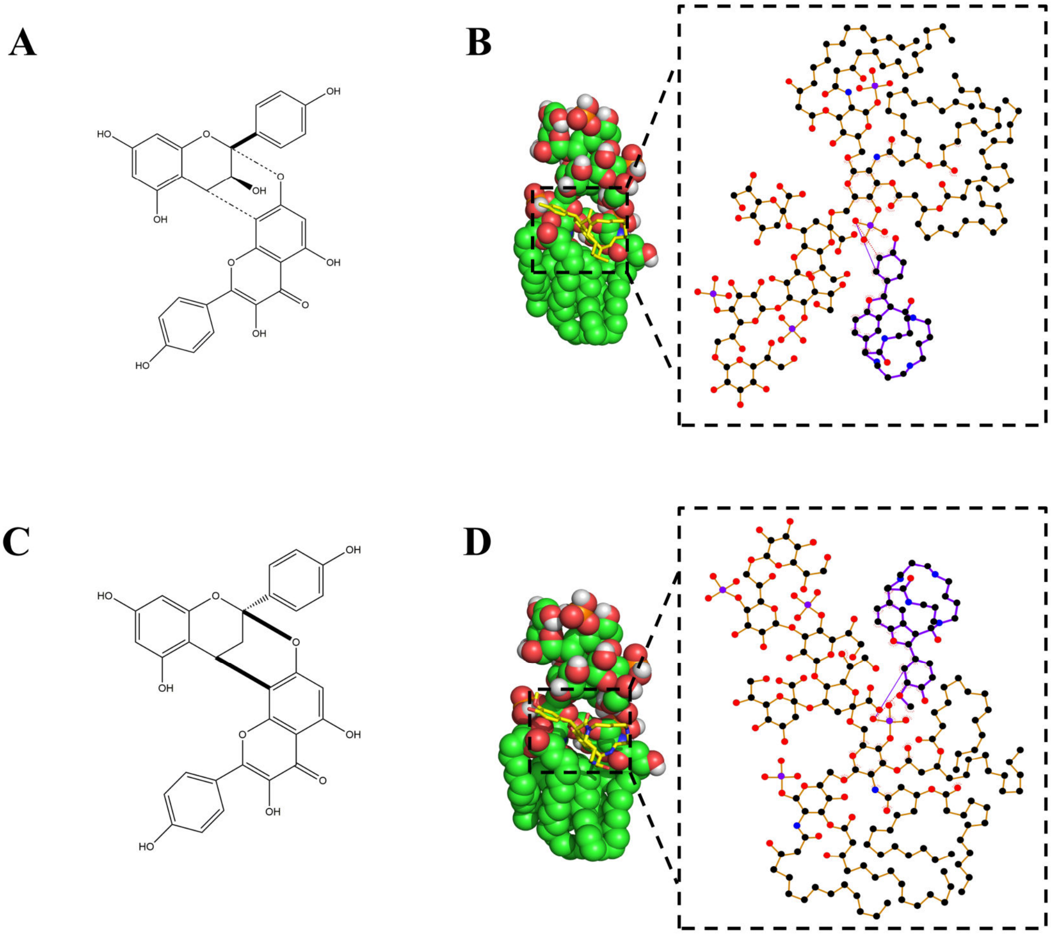

Figure S2. Computer modeling of the LPS and selected natural compounds binding. (A) Two-dimensional structure of Ephedrannin A; (B) The preferred orientation of Ephedrannin A in complex with LPS; (C) Two-dimensional structure of Ephedrannin B. (D) The preferred orientation of Ephedrannin B in complex with LPS. (Black circles mean carbon atoms; red circles mean oxygen atom; blue circles mean nitrogen atoms; purple circles mean phosphorus atoms; purple lines mean covalent bonds; red dash lines mean π-stacking interaction; means hydrophobic contact; means atoms involved in hydrophobic contact).

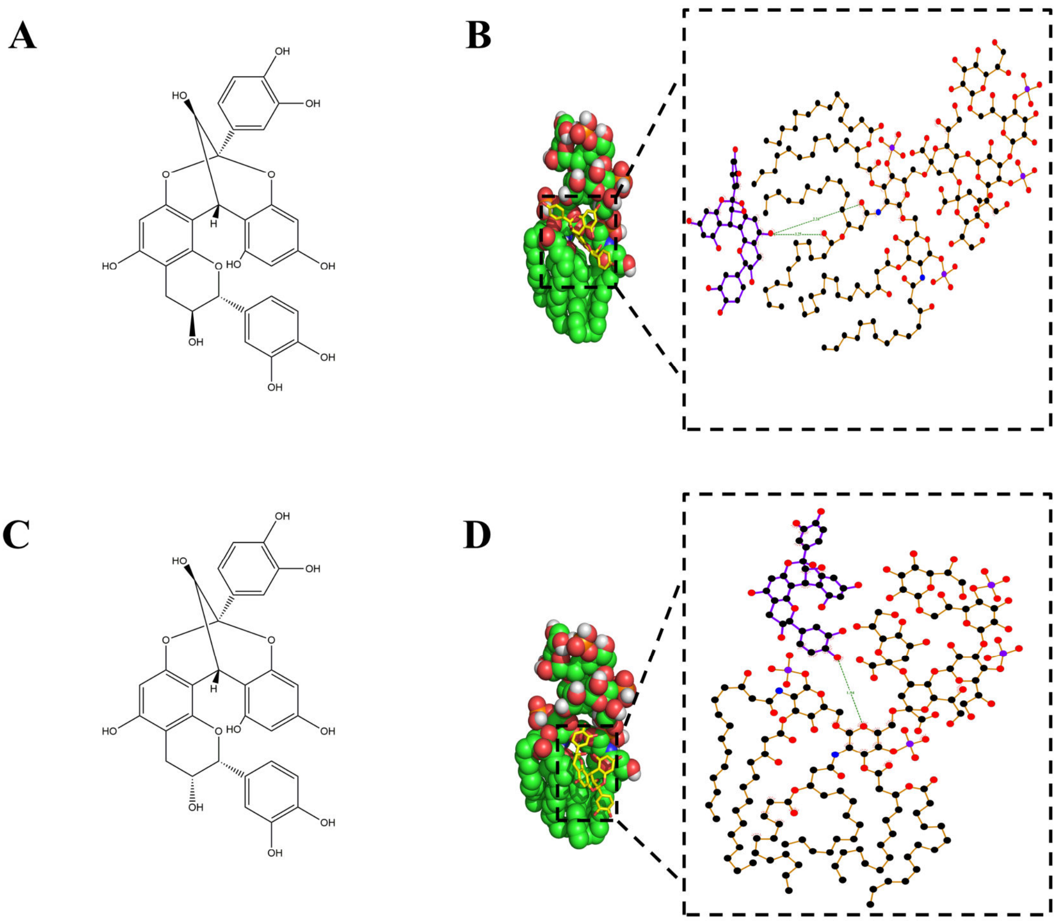

Figure S3. Computer modeling of the LPS and selected natural compounds binding. (A) Two-dimensional structure of Procyanidin A1; (B) The preferred orientation of Procyanidin A1 in complex with LPS; (C) Two-dimensional structure of Procyanidin A2; (D) The preferred orientation of Procyanidin A1 in complex with LPS. (Black circles mean carbon atoms; red circles mean oxygen atom; blue circles mean nitrogen atoms; purple circles mean phosphorus atoms; green dash lines mean hydrogen bonds; means hydrophobic contact; means atoms involved in hydrophobic contact).
